# Design and optimization of wall-climbing robot impeller by genetic algorithm based on computational fluid dynamics and kriging model

**DOI:** 10.1038/s41598-022-13784-z

**Published:** 2022-06-10

**Authors:** Yi Fang, Shuai Wang, Da Cui, Qiushi Bi, Ruihua Jiang, Chuliang Yan

**Affiliations:** 1 School of Mechanical and Aerospace Engineering, Jilin University, Changchun, 130025 China; 2 State Key Laboratory of Internet of Things for Smart City and Department of Electrical and Computer Engineering, University of Macau, Macao, 999078 China

**Keywords:** Engineering, Mechanical engineering

## Abstract

In recent years, wall-climbing robots have begun to replace manual work at heights to reduce economic losses and casualties caused by working at heights. This paper designs a negative pressure adsorption type wall-climbing robot and analyzes the internal fluid movement state of its negative pressure device and the force analysis of the robot when it is adsorbed and balanced. Furthermore, through the experimental prototype, the influence of wall material, robot pose, negative pressure cavity shape and sealing method on the adsorption performance of the wall-climbing robot is explored. The computational fluid dynamics simulation (CFD) simulation method and experimental results are used to verify each other, which proves the correctness of the simulation results. Based on the Kriging surrogate model, the functional relationship between the impeller blade outlet angle, the impeller inlet diameter, the number of blades as the design variables, the negative pressure as the dependent variable was established, and the genetic algorithm (GA) was used to optimize it. Compared with the original design, the optimized design results of impeller parameters have increased the negative pressure value from 3534.75 to 4491.19 Pa, an increase of 27.06%.

## Introduction

With the continuous development of industrial transformation and upgrading and urban modernization, the demand for aerial work in ships, bridges, construction, fire protection, and nuclear energy is also increasing. The future development trend for wall-climbing robots is to replace manual work at heights^1–4^. Gu designed a wall-climbing robot suitable for aircraft fuselage inspection, whose pneumatic system consists of suction cups and cylinder controls^5^. Vega-Heredia has designed a modular window exterior cleaning wall-climbing robot that can transition from one window panel to another^6^. To perform radiation measurements in the high-risk nuclear power plant area instead of a dedicated person, Kim proposed a robot to achieve autonomous driving and climb walls^7^. Stable adsorption capacity is a necessary condition for wall-climbing robots to work efficiently. The main adsorption methods of wall-climbing robots are divided into negative pressure adsorption^8–10^, magnetic adsorption^11,12^, bionic adsorption^13,14^, and electrostatic adsorption^15,16^. Among them, negative pressure adsorption technology research is relatively mature, and the scope of application is broad and not limited by wall materials. Research on the adsorption device of the wall-climbing robot is beneficial to improve the robot's safety performance and operational efficiency and reduce the economic loss and operation risk.

In 1966, A. NISHI et al. designed a wall-climbing robot with a large suction cup as a negative pressure adsorption device. They analyzed the matching of suction cup negative pressure with the performance of the fan. Experiments proved that it could move on a flat wall surface. In 1986, the second-generation prototype was designed, and the safety conditions of the negative pressure device were tested^17–19^. Since then, there have been more and more developments and designs of wall-climbing robots with negative pressure adsorption^20,21^. Liu introduced a wall-climbing robot composed of a vacuum adsorption system and an attachment belt. According to its adsorption principle, a kinematic and dynamic model was established. Under different pressure differential conditions, the vacuum cavity leaked and separated when the robot was adsorbed. The relationship between the ground clearance, the larger the clearance, the greater the air leakage^22^. In order to improve the obstacle-climbing ability of wall-climbing robots, there are more and more researches on rotating-flow negative pressure adsorption devices. Zhao measured the suction characteristics of the suction unit and studied the influence of the wall roughness and shape on the adsorption performance^23^. Amakawa developed a wall-climbing robot attached to the fuselage of an aircraft based on negative pressure technology. Through basic experiments and calculation of the adsorption force, the relationship between the adsorption force generated by the centrifugal fan and the size of the adsorption part, the number of revolutions of the centrifugal fan, the leakage area, and the characteristics of the centrifugal fan and the influence of the curved surface on the suction force are revealed^24,25^. Computational fluid dynamics simulation (CFD) and other methods can be used to analyze, improve and optimize the adsorption device more conveniently^26^. Chen studied the blade vortex's theoretical model, modeled the rotating blade's height, analyzed the secondary flow of the blade by the CFD method, and proposed a blade design method based on the characteristics of the motor^27^. Fallah performed numerical calculations and CFD analysis on the wall-climbing robot designed by his vortex current adsorption to obtain the optimal data and designed a new negative pressure cavity structure^28^. The vortex flow device can form negative air pressure and is also suitable for other fluids. Zhu introduced an electrically-driven underwater whirlpool suction device. The experiments and CFD calculations show that the suction increases quadratically with the increase of speed. Moreover, as the gap increases, the suction first increases and then decreases^29^. Koo gave the static and aerodynamic models of the adsorption mechanism and analyzed them, which provided a basis for design and control^30^. Using a gas pressure sensor to monitor the pressure value in the negative pressure cavity and keeping the pressure in the negative pressure cavity constant can improve the stability of the wall-climbing robot when it moves^31^.

The main contributions of this paper are as follows:

1. In this paper, an experimental prototype of negative pressure adsorption wall-climbing robot is designed. The parameters that affect the adsorption performance of wall-climbing robots are studied, such as wall materials, robot pose, negative pressure cavity shape and sealing method.
2. A new optimization method is proposed for the impeller. The sample points required for the kriging model were obtained by CFD simulation. A surrogate model between impeller parameters and negative pressure values was established using the kriging model. The impeller parameters are optimized based on the genetic algorithm, which improves the adsorption capacity of the robot.

The rest of this article is organized as follows. In “Analysis of fluid state and adsorption conditions” section, the motion state of the fluid in the adsorption device of the wall-climbing robot is analyzed, and the adsorption condition equation is established. In “Adsorption method of wall-climbing robot” section, a prototype of a wall-climbing robot with negative pressure adsorption is designed. In “Prototype experiment and CFD simulation verification” section, the parameters that affect the adsorption performance of the wall-climbing robot are experimentally studied. The CFD method is used for simulation and mutual verification with experiments. In addition, in “Optimized design of impeller” section, the critical components of the impeller are optimized based on the kriging method and GA, and the optimization effect is verified by CFD simulation. Finally, the conclusion is given in “Conclusion” section.

## Analysis of fluid state and adsorption conditions

**Figure 1:**
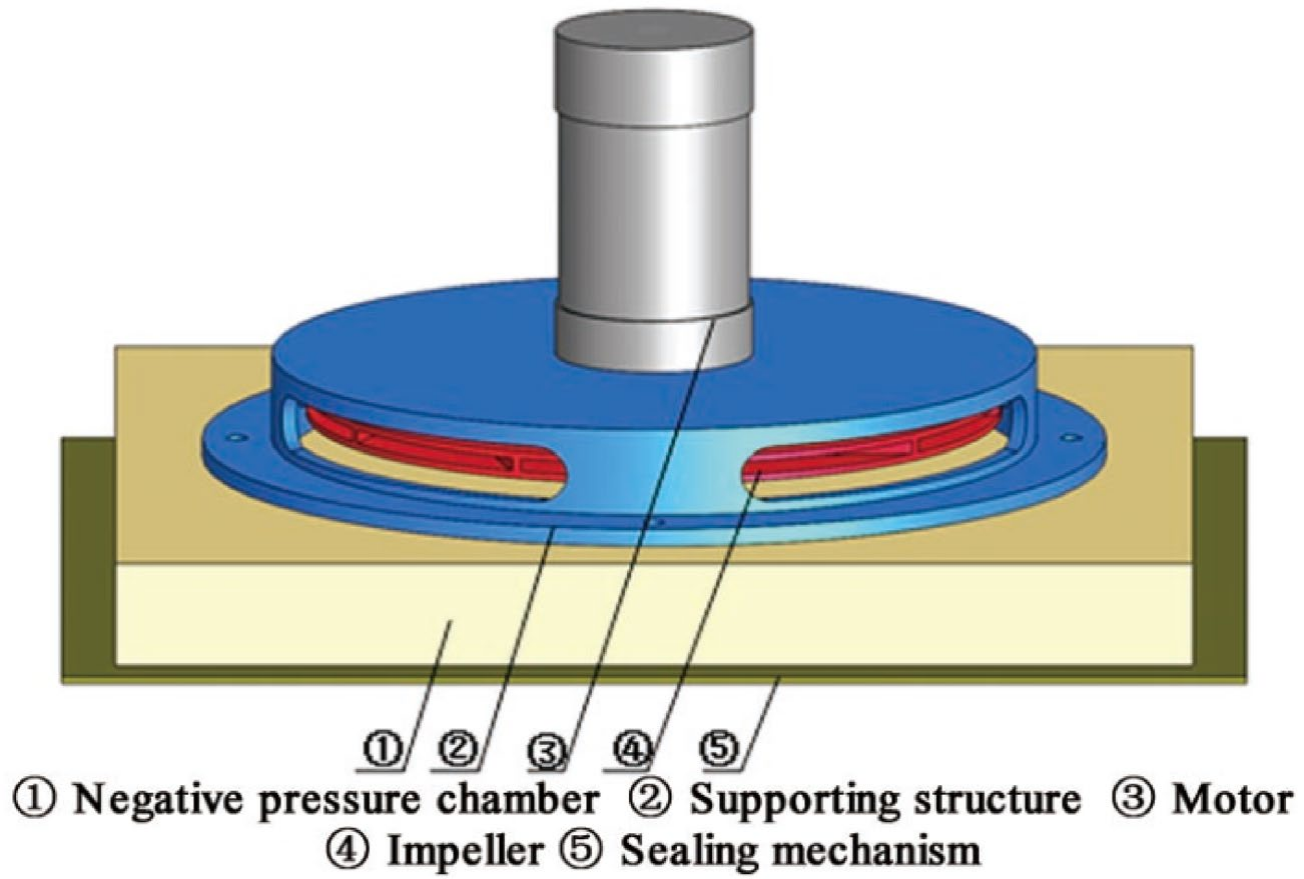
Composition of negative pressure system of wall-climbing robot.

Negative pressure adsorption means that the pressure in a part of the area is lower than the atmospheric pressure. The relatively high-pressure atmosphere flows to the negative pressure area, thereby generating a specific force. If the negative pressure is too small, it will not absorb, resulting in slippage or even falling; when the negative pressure is too enormous, it will make the robot challenging to move and increase the robot's power consumption. Figure 1 shows the components of the negative pressure mechanism of the wall-climbing robot. The motor drives the impeller to rotate at high speed to generate centrifugal force. The impeller draws the gas in the negative pressure cavity, thereby forming a negative pressure in the cavity. The function of the sealing device is to reduce air leakage.

### Analysis of fluid state in negative pressure cavity

#### Analysis of the state of fluid movement in the negative pressure cavity

When analyzing the fluid between the sealing method and the wall gap, the Reynolds number ${\varvec{Re}}$ must first be used to determine whether the fluid flow is laminar or turbulent. When $\user2{Re < }2320$, the fluid motion was laminar; then $\user2{Re > }2320$, the fluid motion was turbulent^32^.

The calculation formula of Reynolds number ${\varvec{Re}}$ is:

1 $$Re = \frac{{vd_{k} }}{\eta }$$

where $v$ is the average flow velocity of the fluid; $d_{k}$ is the hydraulic diameter of the flow section; $\eta$ is the kinematic viscosity of the fluid, and $\eta { = }\mu /\rho$, $\mu$ is the dynamic viscosity of the fluid, $\rho$ is the density of the fluid, at room temperature, $\eta { = }1.57 \times 10^{ - 5} {\text{ m}}^{{2}} {\text{/s}}$.

According to the similar principle, the hydraulic diameter of non-standard structure pipeline can be calculated by the following formula^33^

2 $$d_{k} = 4\frac{A}{S}$$

Among them, $A$ is the cross-sectional area of the flow; $S$ is the sum of the inner and outer perimeters of the flow.

**Figure 2:**
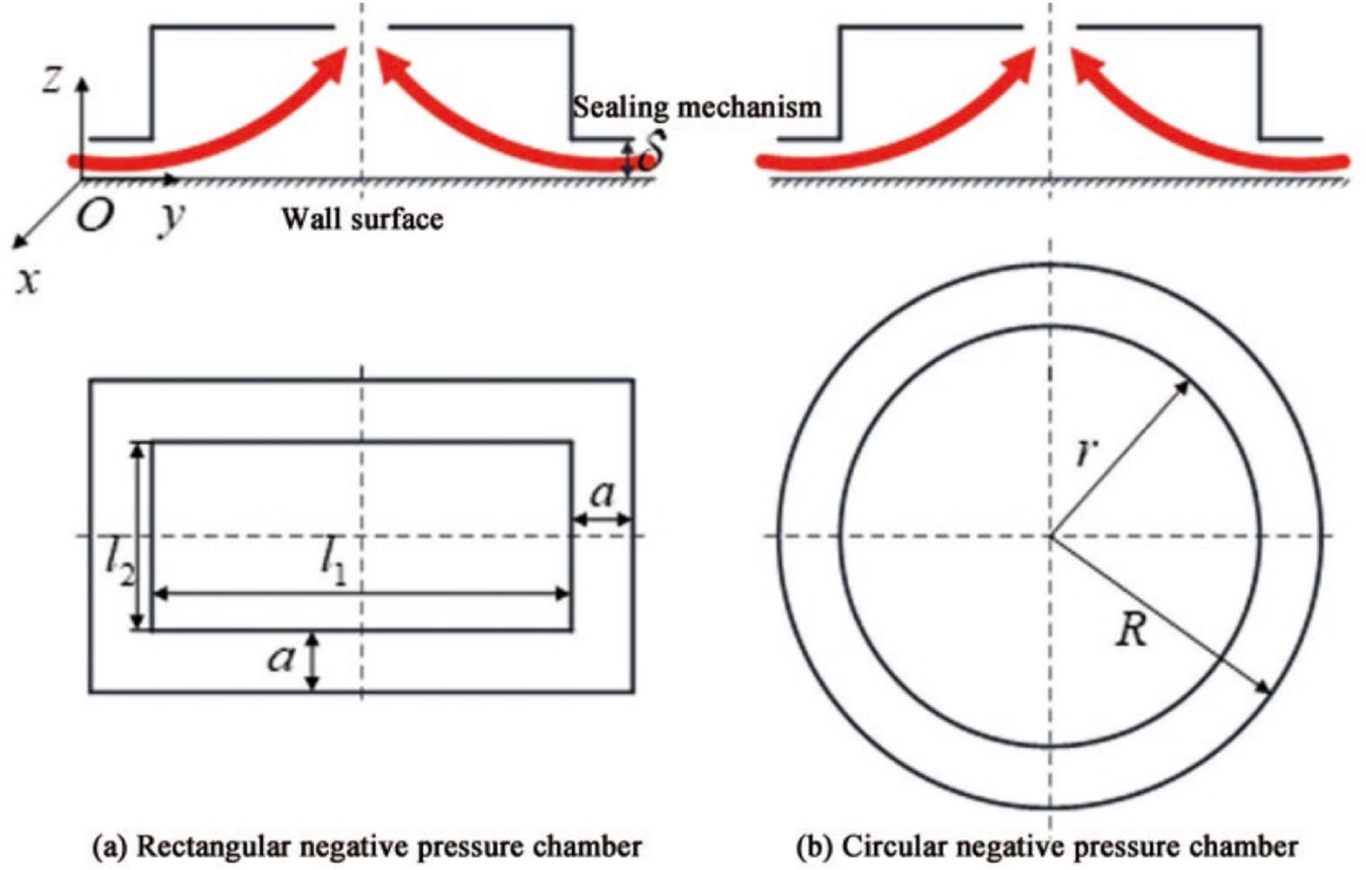
Structure model of negative pressure cavity.

The negative pressure cavity structure of the negative pressure adsorption type wall-climbing robot has a rectangle and a circle, and the structures are shown in Fig. 2a and b, respectively.

As shown in Fig. 2a, for the rectangular negative pressure cavity, the hydraulic diameter $d_{ks}$ between the sealing device and the wall gap $\delta$ is:

3 $$d_{ks} = 4\frac{A}{S}{ = }4\frac{{\left( {l_{1} + 2a} \right)\left( {l_{2} + 2a} \right) - l_{1} l_{2} }}{{2\left( {l_{1} + l_{2} } \right) + 2\left( {l_{1} + 2a + l_{2} + 2a} \right)}} = \frac{{2a\left( {l_{1} + l_{2} } \right) + 4a^{2} }}{{l_{1} + l_{2} + 2a}}$$

Among them, $l_{1}$ is the dimension of the negative pressure cavity in the $y$ direction; $l_{2}$ is the dimension of the negative pressure cavity in the $x$ direction; $a$ is the side length of the sealing method.

Take $a = 0.1l_{1} = 0.1l_{2}$, then formula (3) can be simplified to $d_{ks} \approx 0.2l_{1}$, considering that the wall-climbing robot is generally small in size, taking $l_{1} \le 0.5{\text{ m}}$, then the rectangular negative pressure cavity $d_{ks} \le 0.1{\text{ m}}^{{ - 1}}$, the flow velocity between the sealing method and the wall gap is very low, taking $v \le 0.1{\text{ m/s}}$. From Eq. (1), the Reynolds number ${\varvec{Re}}_{{\varvec{s}}} \le 637 < 2320$ of the rectangular negative pressure cavity can be obtained. Therefore, the flow of fluid between the sealing method of the rectangular negative pressure cavity and the gap in the wall is laminar flow.

In the same way, the hydraulic diameter $d_{kc}$ of the circular negative pressure cavity is:

4 $$d_{kc} = 4\frac{A}{S} = 4\frac{{\pi R^{2} - \pi r^{2} }}{2\pi R + 2\pi r} = 2\left( {R - r} \right) \le 0.1{\text{ m}}$$

Therefore ${\varvec{Re}}_{c} \le 637 < 2320$, the flow between the sealing method of the fluid-shaped negative pressure cavity and the gap in the wall is also laminar.

#### Force analysis of fluid movement in negative pressure cavity

Since the air velocity entering the negative pressure cavity through the gap between the sealing method and the wall is shallow and laminar, the Navier–Stokes equation (N–S equation) can be obtained:

5 $$\rho_{air} \frac{du}{{dt}} = - \nabla p + \mu_{air} \nabla^{2} u + \rho_{air} f$$

Among them, $\rho_{air}$ is the density of air, at room temperature (20 °C), its value is 1.20 kg/m^3^; $u$ is the fluid velocity, and its components in the $x$, $y$ and $z$ directions are $u_{x}$, $u_{y}$ and $u_{z}$ respectively, $\nabla$ is the gradient symbol; *p* is the pressure between the sealing device and the wall gap $\delta$; $\mu_{air}$ is the dynamic viscosity of the air, at room temperature (20 °C), its value is $0.0183 \times 10^{ - 3}$ Pa·s; $f$ is the unit mass force, and its components in the $x$, $y$ and $z$ directions are $f_{x}$, $f_{y}$ and $f_{z}$ respectively.

Assuming that all the air particles in the gap are moving in parallel and straight lines, the speed $u$ has nothing to do with $x$, $y$ direction, that is $u = u\left( z \right)$^34^.

Choose the $y$ axis direction as the air movement direction, only $u_{y}$ non-zero and $\partial u/\partial y = 0$, $u_{x}$ and $u_{z}$ are both 0, the N-S equation in the and directions is

6 $$0{ = } - \frac{\partial p}{{\partial x}},0 = - \frac{\partial p}{{\partial z}}$$

Then the pressure $p$ has nothing to do with $x$, $z$, that is $p = p\left( y \right)$;

Assuming that the air in the gap is not compressible, there are:

7 $$\left\{ \begin{gathered} \nabla \user2{u = }\frac{{\partial u_{x} }}{\partial x} + \frac{{\partial u_{y} }}{\partial y} + \frac{{\partial u_{z} }}{\partial z} = 0 \hfill \\ f_{x} = f_{y} = f_{z} = 0 \hfill \\ \end{gathered} \right.$$

Substituting Eqs. (6) and (7) into Eq. (5), we can get:

8 $$\frac{{d^{2} u}}{{d^{2} z}} = \frac{1}{{\mu_{air} }}\frac{dp}{{dy}}$$

Performing quadratic integration on formula (8) can be obtained:

9 $$u = \frac{1}{{\mu_{air} }}\frac{dp}{{dy}}\left( {\frac{{z^{2} }}{2}} \right) + Cz + D$$

Among them, C and D are integral constants, and the boundary conditions are:

10 $$\left\{ \begin{gathered} z = 0,u = 0 \hfill \\ z = \delta ,u = 0 \hfill \\ \end{gathered} \right.$$

Substituting Eq. (10) into Eq. (9), we can get:

11 $$u = \frac{1}{{2\mu_{air} }}z\left( {z - \delta } \right)\frac{dp}{{dy}}$$

The air flow $q$ into the negative pressure cavity through the gap is

12 $$q = 2\int_{0}^{\delta } {u\left( {l_{1} + 2a} \right)} dz + 2\int_{0}^{\delta } {u\left( {l_{2} + 2a} \right)} dz = \frac{{\delta^{3} }}{{6\mu_{air} a}}\left( {p_{0} - p_{1} } \right)\left( {l_{1} + l_{2} + 4a} \right)$$

**Figure 3:**
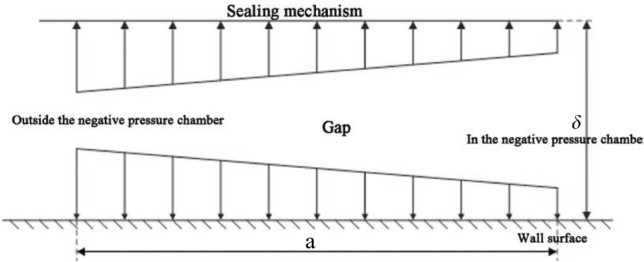
Pressure distribution in the gap.

Among them, $p_{0}$ is the standard atmospheric pressure value, which is 101.325 kPa; $p_{1}$ is the internal pressure of the negative pressure cavity of the wall-climbing robot, and the pressure distribution in the gap is shown in Fig. 3.

It can be seen from Fig. 3 that the air pressure outside the negative pressure cavity is $p_{0}$, and the pressure becomes $p_{1}$, after passing through the gap, resulting in a linear pressure distribution with a pressure difference of $\Delta p = p_{0} - p_{1}$, then the resultant force $F_{\delta }$ of the atmospheric pressure acting on the sealing method is:

13 $$F_{\delta } { = }\frac{{p_{0} - p_{1} }}{2}2a\left( {l_{1} + 2a + l_{2} + 2a} \right) = a\left( {p_{0} - p_{1} } \right)\left( {l_{1} + l_{2} + 4a} \right)$$

The pressure $F_{p}$ inside the negative pressure cavity is:

14 $$F_{p} = \left( {p_{0} - p_{1} } \right)l_{1} l_{2}$$

Make:

15 $$\frac{{a\left( {l_{1} + l_{2} + 4a} \right)}}{{l_{1} l_{2} }}{ = }E$$

The result is:

16 $$F_{\delta } { = }EF_{p}$$

### Adsorption conditions of wall-climbing robot

**Figure 4:**
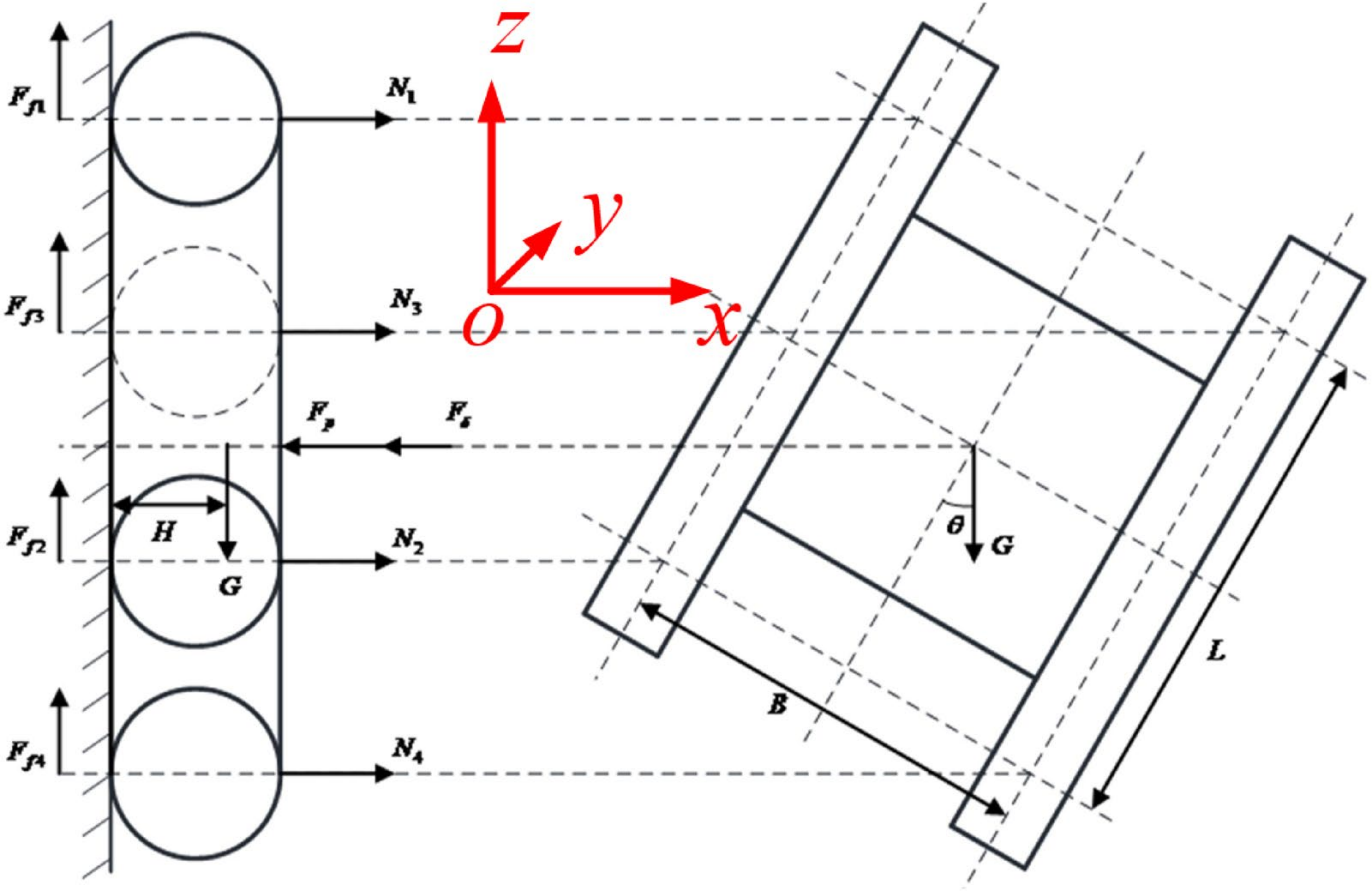
Force analysis of wall-climbing robot at any posture.

It is assumed that the supporting force of the wall acting on the robot is borne by the four rollers on both sides of the track, *yoz* is the wall, the *z*-axis is the forward direction of the robot, and the *x*-axis is perpendicular to the wall. Figure 4 shows the force analysis diagram of the wall-climbing robot in any posture. Among them, $N_{1}$, $N_{2}$, $N_{3}$ and $N_{4}$ are the pressure of the wall on the left front wheel, left rear wheel, right front wheel and right rear wheel of the wall-climbing robot respectively; $F_{f1}$, $F_{f2}$, $F_{f3}$ and $F_{f4}$ are the static friction forces of the wall on the left front wheel, left rear wheel, right front wheel and right rear wheel of the wall-climbing robot, respectively, $F_{f1} = \mu_{1} N_{1}$, $F_{f2} = \mu_{1} N_{2}$, $F_{f3} = \mu_{1} N_{3}$ and $F_{f4} = \mu_{1} N_{4}$.

$\mu_{1}$ is the static friction coefficient between the wall and each wheel of the wall-climbing robot; $G$ is the gravity of the wall-climbing robot, and its magnitude is $G = mg$; $m$ is the mass of the wall-climbing robot; $g$ is the acceleration of gravity; $H$ is the distance between the center of gravity of the wall-climbing robot and the wall; $L$ is the distance between the front and rear wheels of the robot; $B$ is the gauge of the two crawlers of the wall-climbing robot; $\theta$ is the angle between the gravity and the orientation of the body.

The robot is balanced by forces in the x and z directions and moments around the two outermost rollers. The dynamic equation of the wall-climbing robot in any posture is:

17 $$\left\{ \begin{gathered} N_{1} + N_{2} + N_{3} + N_{4} - F_{p} - F_{\delta } = 0 \hfill \\ F_{f1} + F_{f2} + F_{f3} + F_{f4} - G = 0 \hfill \\ \left( {F_{p} + F_{\delta } } \right)\frac{L}{2} - N_{1} L - N_{3} L - GH\cos \theta = 0 \hfill \\ \left( {F_{p} + F_{\delta } } \right)\frac{B}{2} - N_{3} B - N_{4} B - GH\sin \theta = 0 \hfill \\ N_{1} + N_{4} { = }N_{2} { + }N_{3} \hfill \\ \end{gathered} \right.$$

From Eq. (17), it can be seen that under the condition of any pose of the wall-climbing robot body, the differential pressure $F_{p}$ is:

18 $$\left\{ \begin{gathered} F_{p} = \frac{G}{{\left( {1 + E} \right)\mu_{1} }} \hfill \\ F_{p} = \frac{2GH}{{1 + E}}\left( {\frac{\cos \theta }{L} + \frac{\sin \theta }{B}} \right) + 4N_{3} \hfill \\ \end{gathered} \right.$$

In order to ensure the normal work of the wall-climbing robot, it needs $N_{3}$ to be greater than 0, which can be obtained from Eq. (18). Under the condition of any posture of the robot body, the adsorption condition of the wall-climbing robot is:

19 $$F_{p} > \max \left\{ {\frac{G}{{\left( {1 + E} \right)\mu_{1} }},\frac{2GH}{{1 + E}}\left( {\frac{\cos \theta }{L} + \frac{\sin \theta }{B}} \right)} \right\}$$

## Adsorption method of wall-climbing robot

### Design of wall-climbing robot and its impeller model

Table 1 shows the basic parameters of the designed wall-climbing robot.

**Table 1:** Basic parameters of wall-climbing robot.

Parameter	Symbol	Value	Unit
Distance between front and rear wheels	*L*	200	mm
Track gauge on both sides	*B*	300	mm
The height of the center of gravity of the robot	*H*	40	mm
The mass of the robot	*m*	2.5	kg
The load quality of robot	*m*_*d*_	2.5	kg
The length of negative pressure cavity	*l*_*1*_	250	mm
The width of the negative pressure cavity	*l*_*2*_	200	mm
The width of the sealing device	*a*	10	mm

**Figure 5:**
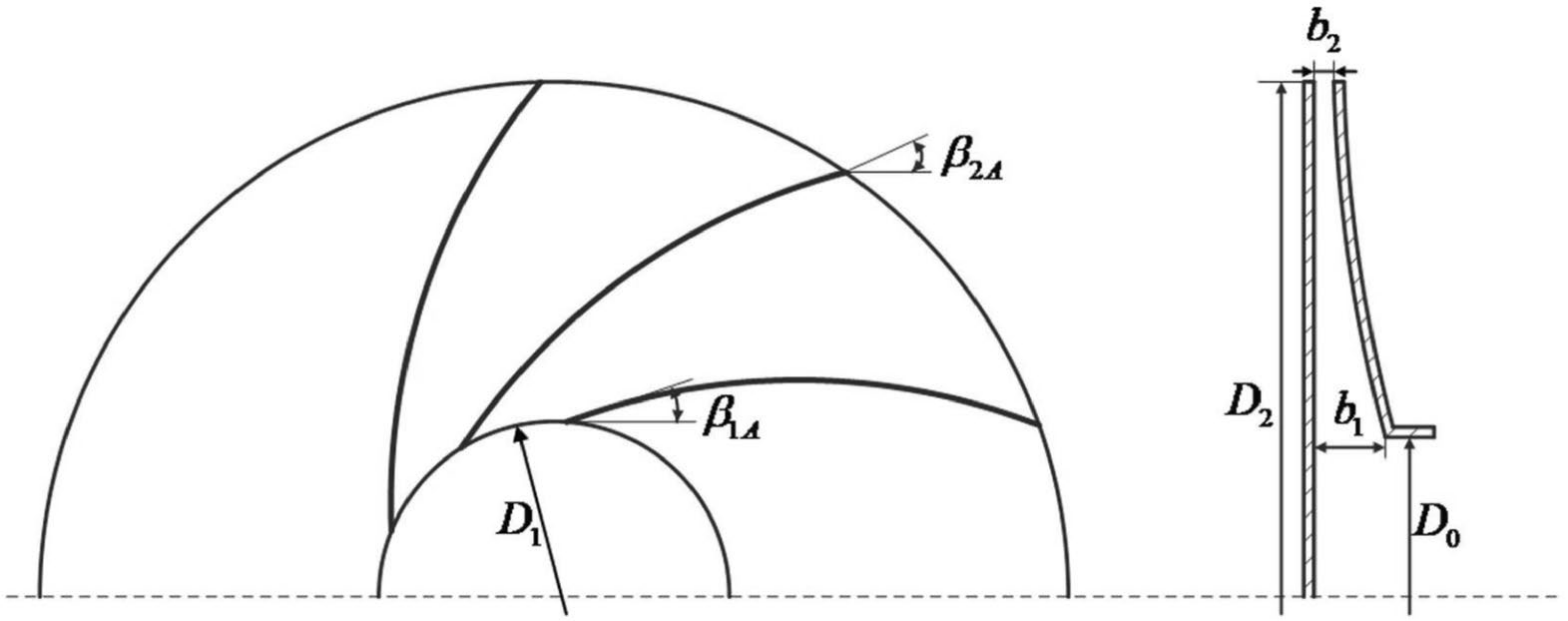
Main parameters of impeller.

We need to design the appropriate blade size according to the load requirements of the robot. Therefore, the load quality of the robot in Table 1 refer to the pre-assumed impeller design conditions. The structure of the impeller plays a vital role in the size of the negative pressure. The main parameters of the impeller include impeller inlet diameter $D_{0}$, blade inlet diameter $D_{1}$, impeller outlet diameter $D_{2}$, blade inlet size $b_{1}$, blade outlet size $b_{2}$, blade inlet geometric angle $\beta_{1A}$ and blade outlet geometric angle $\beta_{2A}$^35^, as shown in Fig. 5.

**Figure 6:**
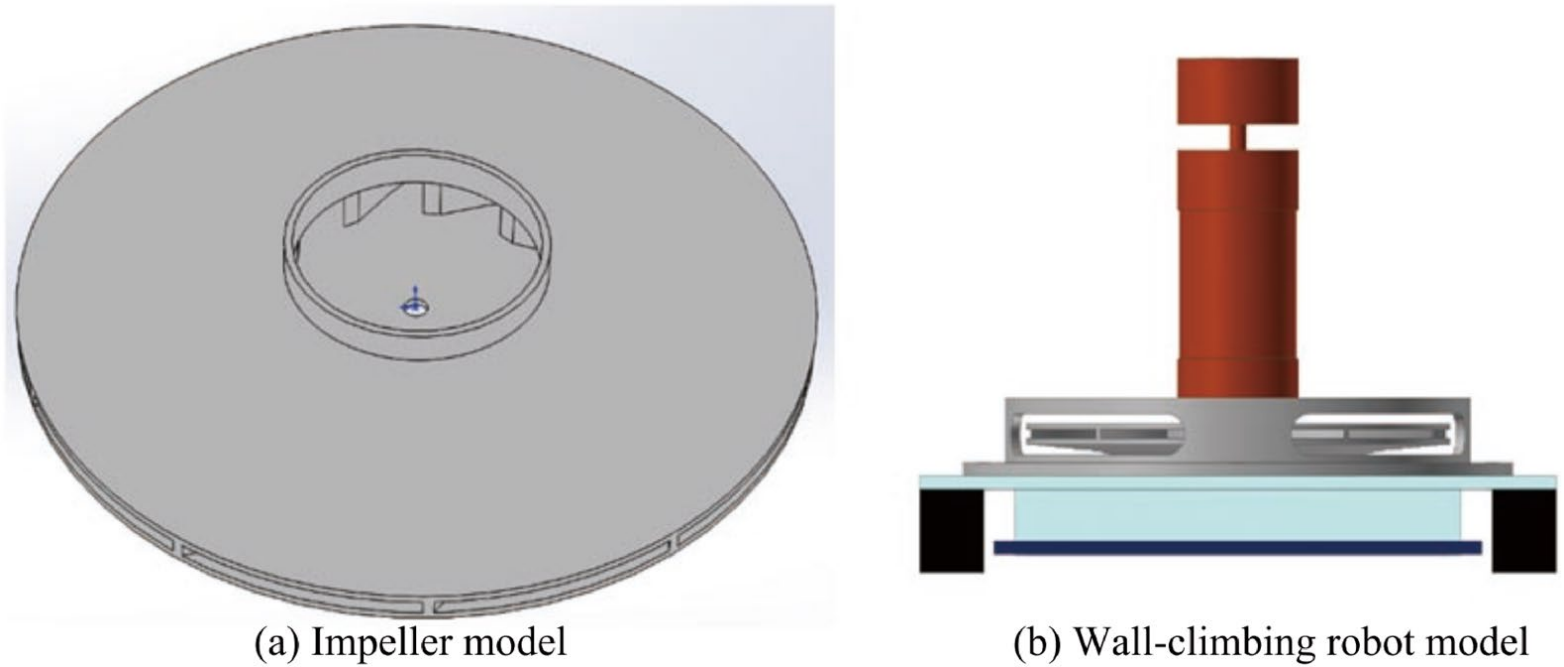
Wall-climbing robot and its impeller model.

According to the minimum adsorption pressure under the adsorption equilibrium condition and the parameter design method of the impeller, the main parameters of the impeller can be obtained as shown in Table 2^36^, the three-dimensional model of the impeller established according to the impeller parameters is shown in Fig. 6a, and the wall-climbing robot model is shown in Fig. 6b. The closed impeller consists of a wheel cover, blades, and a wheel disc. The closed structure reduces internal leakage losses, so the working efficiency is higher than that of open and semi-open impellers, suitable for high-pressure situations.

**Table 2:** Main parameters of impeller.

Parameter	Symbol	Value	Unit
Blade inlet angle	$\beta_{1A}$	30	°
Blade outlet angle	$\beta_{2A}$	50	°
Impeller inlet diameter	$D_{0}$	62	mm
Blade inlet diameter	$D_{1}$	68	mm
Impeller outlet diameter	$D_{2}$	200	mm
Blade inlet width	$b_{1}$	13	mm
Blade outlet width	$b_{2}$	4	mm
Blade number	$Z$	10	pcs

### Experimental prototype of wall-climbing robot

**Figure 7:**
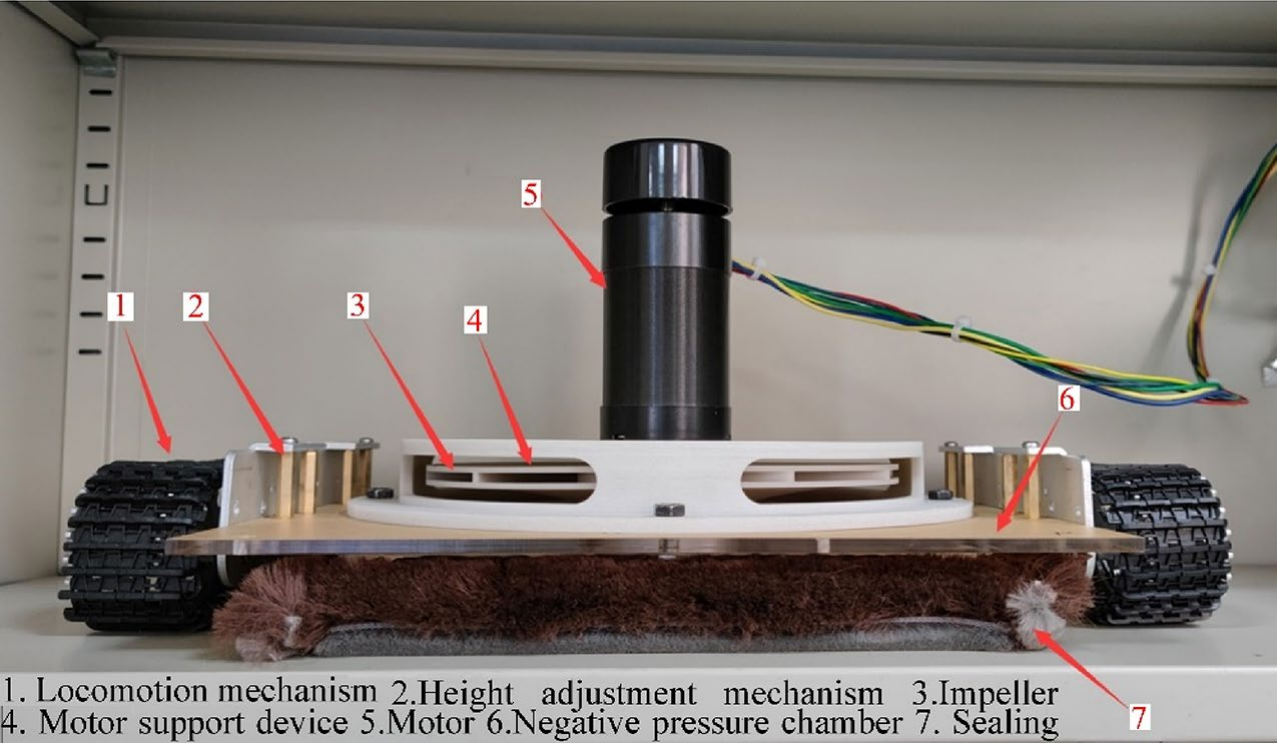
Experimental prototype of wall-climbing robot.

The actual object designed according to the above-mentioned three-dimensional model parameters of the wall-climbing robot is shown in Fig. 7. The support structure between the impeller and the motor is made of high-strength nylon glass fibre 3D printing, which can support the motor and prevent the impeller from eccentricity during operation. The ground clearance of the negative pressure cavity can be adjusted by replacing the studs of different lengths in the crawler structure. The influence of different ground clearance of the negative pressure cavity on the adsorption performance of the wall-climbing robot can be adjusted be analyzed. The main structure of the negative pressure cavity is made of high-strength acrylic plate material, which ensures the structural strength of the negative pressure cavity and facilitates manufacturing. The sealing device uses the sealing top produced by the 3 M company, which can reduce air leakage and achieve stable air pressure in the negative pressure cavity. The air pressure sensor is arranged for real-time measurement of the pressure inside the negative pressure cavity.

## Prototype experiment and CFD simulation verification

### Prototype experiment and analysis of its results

In order to control variables and avoid accidental errors, the speed of the driving motor of the impeller during the experiment is 8000 r/min. Furthermore, all the working condition experiments are repeated three times, and the final experimental value obtained is the average of the three experimental results.

#### The effect of wall material

**Figure 8:**
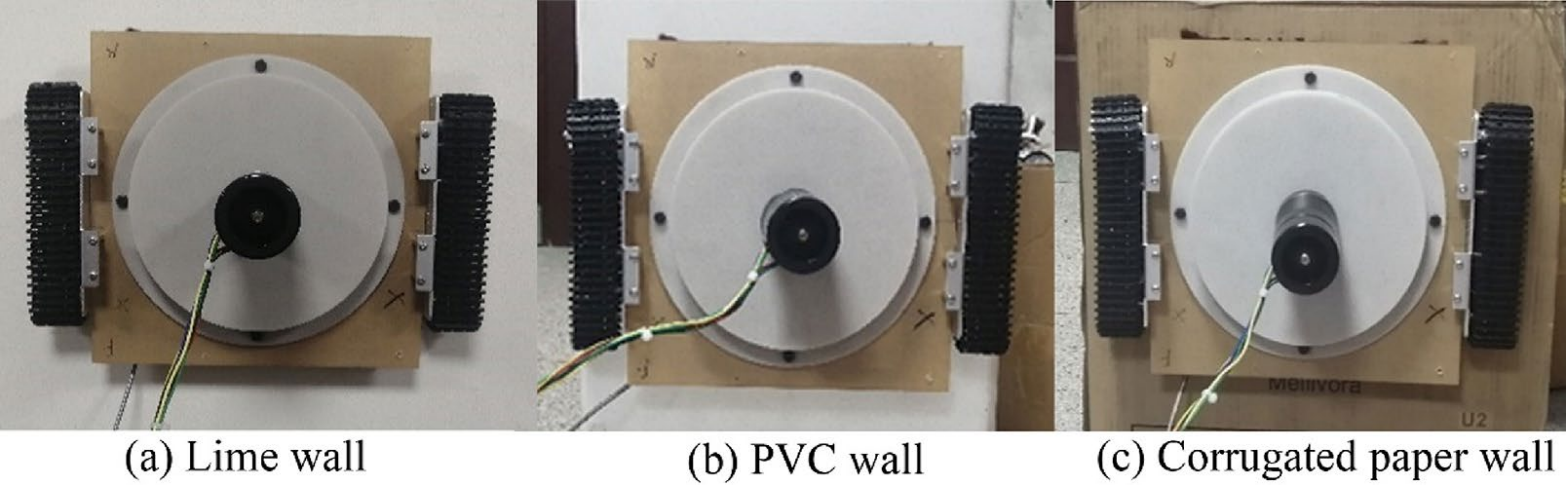
Experiments on different wall materials.

In order to explore the adsorption performance of the wall-climbing robot when adsorbing on different wall materials, three different wall materials were used to conduct adsorption experiments. As shown in Fig. 8, the robot is adsorbed on the lime wall, the PVC (Poly Vinyl Chloride) wall and the ordinary corrugated box wall. The walls of lime, PVC and corrugated boxes have obvious grades in terms of hardness and surface roughness, and can have obvious contrast effects. In addition, it is easier to experiment with walls of these three materials in a laboratory environment.

The experiment measured that the negative pressure value on the lime wall is 3351.24 Pa, the negative pressure value on the PVC wall surface is 3880.0 Pa, and the negative pressure value on the ordinary corrugated box wall surface is 4404.36 Pa. When the suction device of the robot is working, under the action of negative pressure, both the PVC material wall and the ordinary corrugated box wall are deformed. The deformation of the corrugated paper wall is more significant than that of the PVC material surface, which causes the gap between the negative pressure cavity and the wall to shrink. The air leakage between the gaps is reduced, thereby increasing the negative pressure value. At the same time, the lime wall surface has almost no deformation, and the gap between the negative pressure cavity and the wall surface has no noticeable change, so the negative pressure value is the smallest. When the wall deformation is not considered, the wall material has little effect on the adsorption performance.

#### The influence of robot orientations

**Figure 9:**
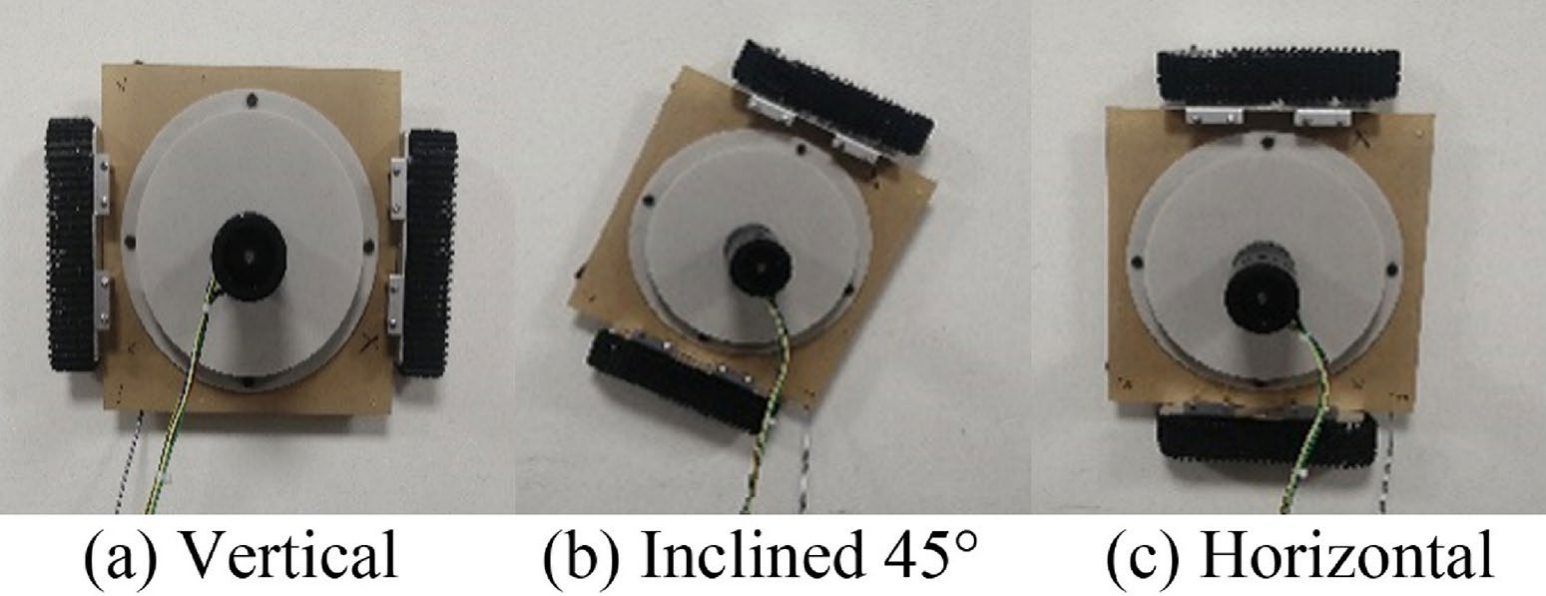
Three orientations of the robot.

Figure 9 shows the experimental results when the robot is adsorbed on the same lime wall in three different orientations, including three different robot orientations: vertical, inclined 45°, and horizontal. According to experiments, the negative pressure values of the wall-climbing robot in the three states are 3531.24 Pa, 3557.76 Pa, and 3578.16 Pa, respectively. The difference between the three experimental conditions is negligible. It can be considered that the posture of the wall-climbing robot adsorbed on the wall is opposite. The negative pressure value has no effect.

#### The influence of the shape of the negative pressure cavity

**Figure 10:**
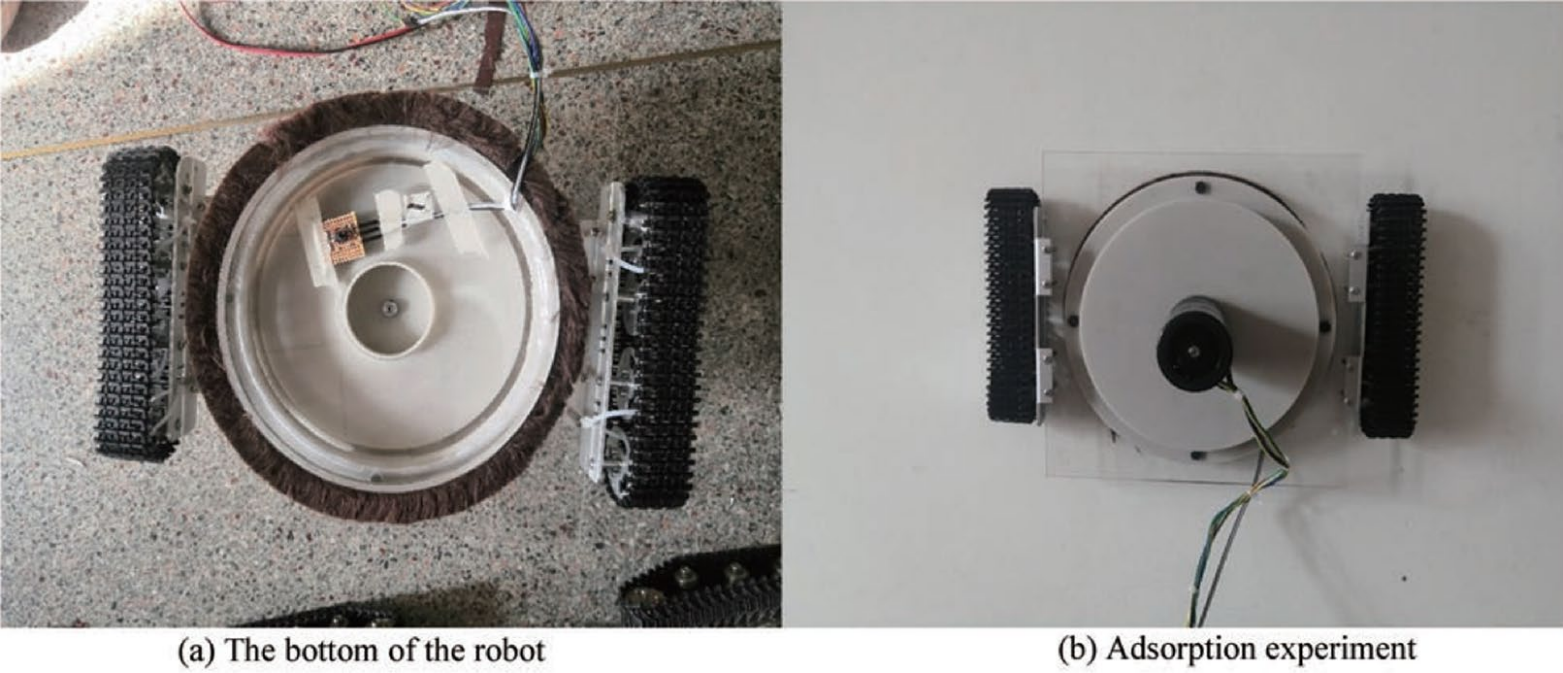
Circular negative pressure cavity and experiment.

To compare the effects of different negative pressure cavity shapes on the adsorption performance, a circular negative pressure cavity (0.05 m^2^) with the same area as the rectangular negative pressure cavity was used for adsorption performance experiments. Figure 10 shows the experiment of a wall-climbing robot in which the circular negative pressure cavity is adsorbed on the lime wall. The experiments were carried out with three robot orientations consistent with the rectangular negative pressure device to control the variables to reduce the influence of other factors on adsorption. Table 3 lists the negative pressure values of two different cavity types. It can be seen that compared with the rectangular negative pressure cavity, the performance of the circular negative pressure cavity is better because the gas flowability of the circular negative pressure cavity is better.

**Table 3:** Negative pressure values of different cavity types.

Negative pressure cavity shape	Vertical direction (Pa)	Tilt 45° direction (Pa)	Horizontal direction (Pa)
The rectangular	3531.24	3557.76	3578.16
The circular	4075.52	4066.24	4080.66

#### Influence of sealing device

**Figure 11:**
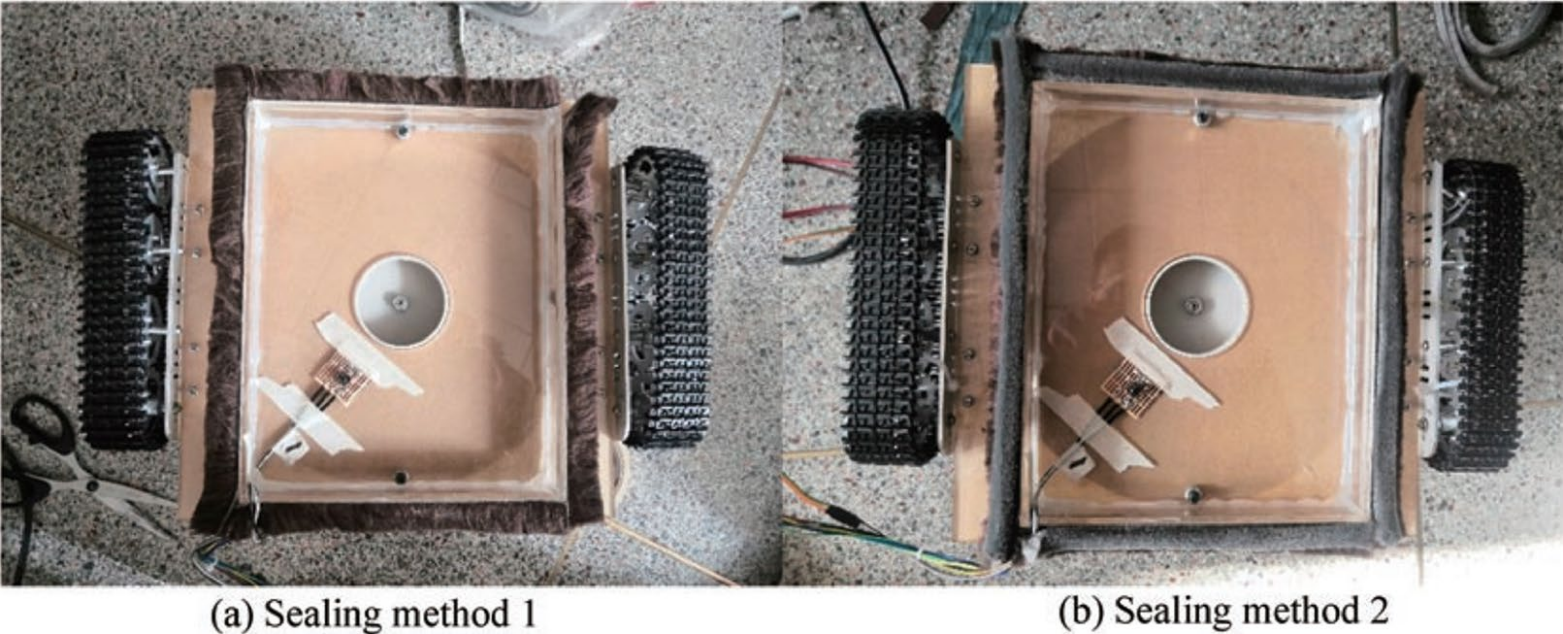
Two sealing methods.

Two different negative pressure cavity sealing methods are shown in Fig. 11. The sealing method is shown in Fig. 11a used a sealing skirt to surround the bottom of the negative pressure cavity. The sealing method is shown in Fig. 11b is based on the first seal and then adds a layer to the bottom of the negative pressure cavity. The sealing skirt reduces the ground clearance of the robot. Two sealing methods are used to conduct experiments with the robot body upright. The experimental results are: the negative pressure value of the first sealing method is 3531.24 Pa, and the negative pressure value of the second sealing method is 3720.96 Pa. It can be seen that the second sealing method reduces the air leakage flow when the robot is adsorbing and increases the negative pressure value by 5.4%. Therefore, a reasonable sealing structure is a key to the regular operation of the wall-climbing robot's adsorption device.

In summary, when designing the suction device of the wall-climbing robot, it is essential to consider the shape of the negative pressure cavity and the structure of the sealing method to improve the suction performance of the wall-climbing robot.

### CFD simulation and verification

#### CFD setup and simulation

**Figure 12:**
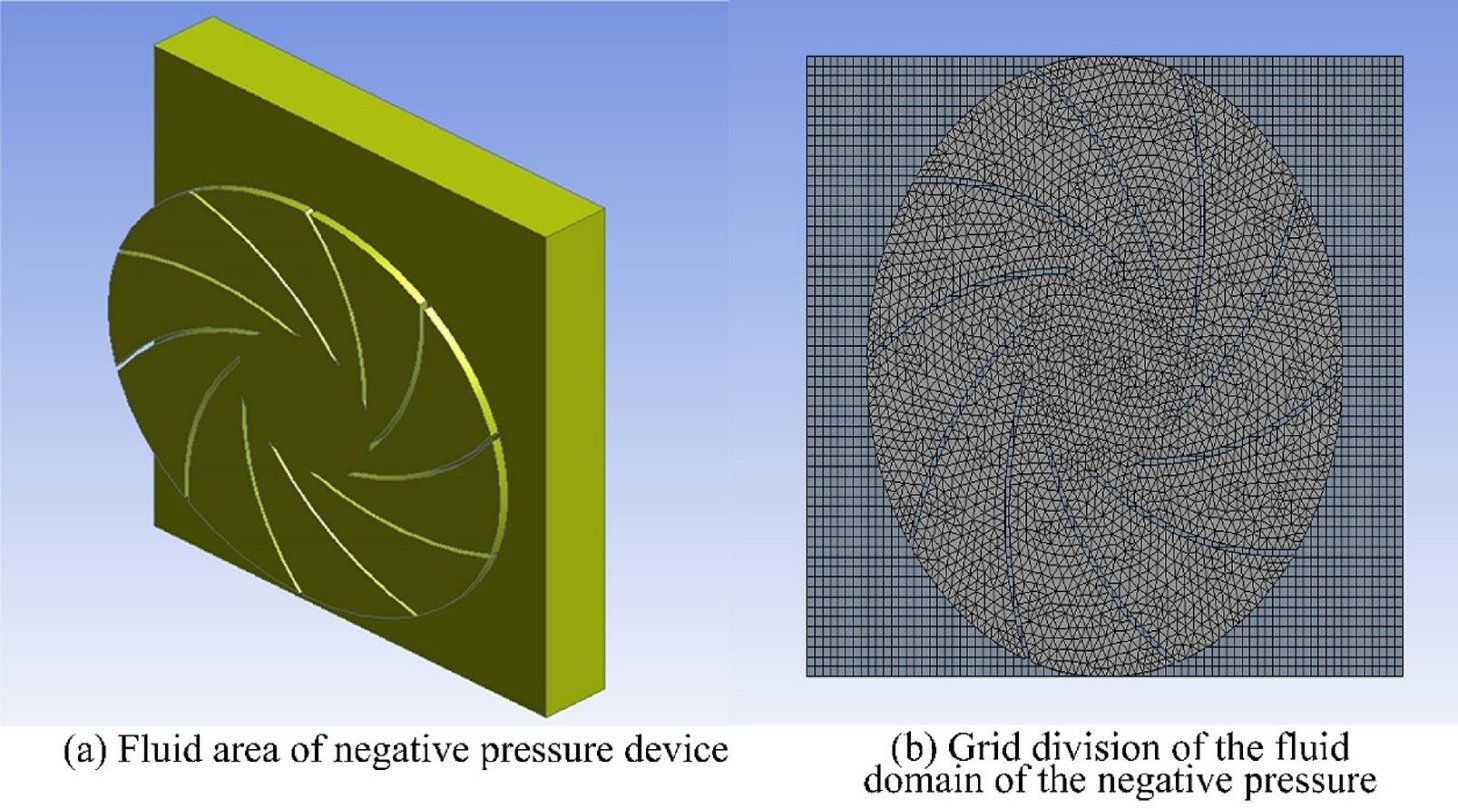
Grid division of negative pressure device.

In order to compare and analyze the experimental results, the CFD method is used to simulate and analyze the fluid movement inside the negative pressure device when the wall-climbing robot is working. The grid division of the fluid domain of the negative pressure device is shown in Fig. 12. The fluid domain mesh of the negative pressure device is mainly composed of tetrahedral and hexahedral mesh. The tetrahedral mesh is primarily located in the impeller fluid domain mesh. The hexahedral mesh is mainly located in the fluid domain mesh in the negative pressure cavity.

**Figure 13:**
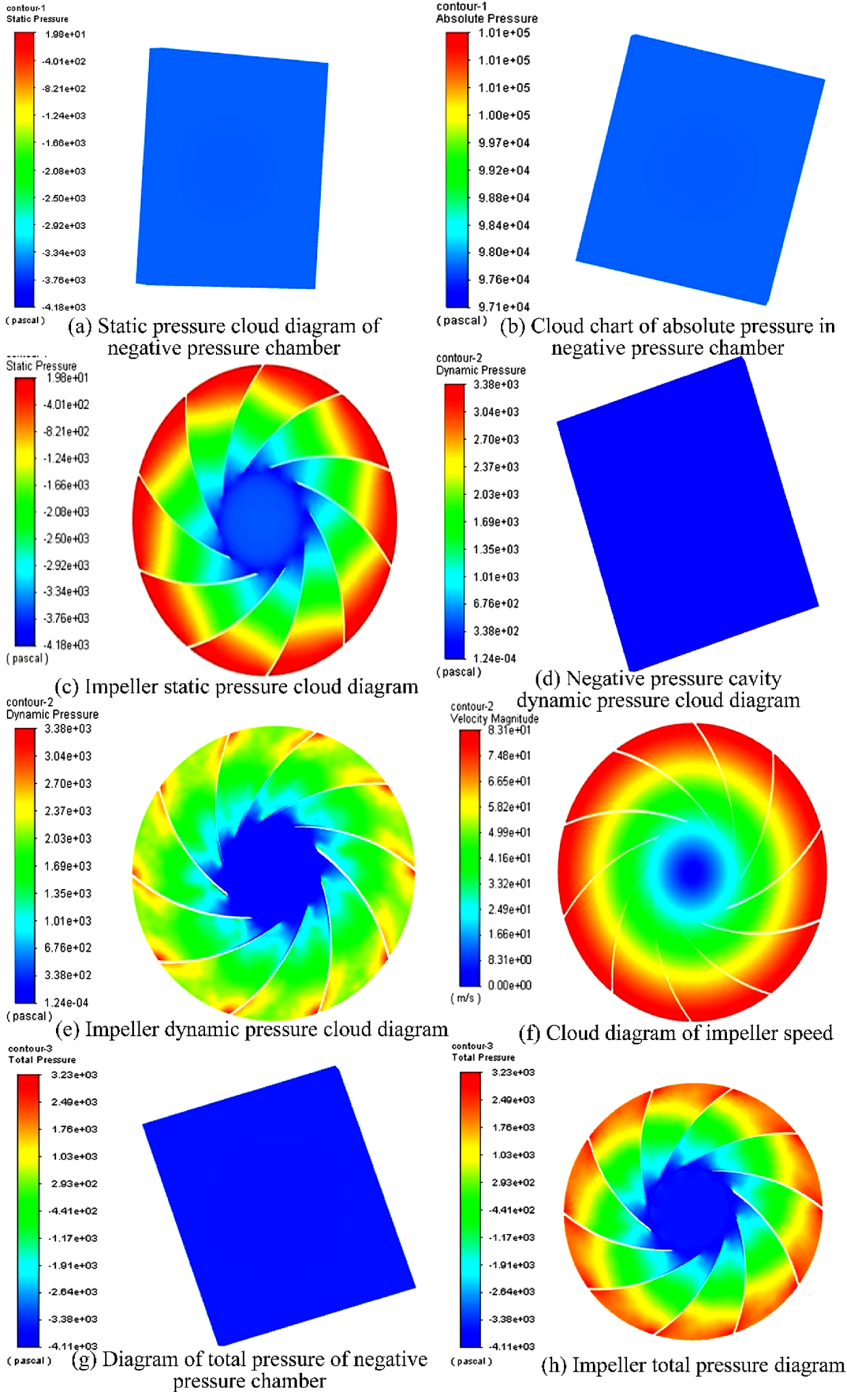
Pressure cloud diagram of impeller and negative pressure cavity.

Figure 13 shows the simulated stress cloud diagram of the impeller and the negative pressure cavity. Figure 13a and b, respectively, are the static pressure cloud diagram and the absolute pressure cloud diagram of the negative pressure cavity. The two stress distributions are the same because the static pressure $p_{sta}$ in Fluent refers to the difference between the absolute pressure $p_{abs}$ and the operating pressure $p_{ope}$. The operating pressure set in the simulation is the standard atmospheric pressure 101,325 Pa. The pressure value in the static pressure cloud chart is negative, as shown in Fig. 13a. The negative pressure value is about 3500 Pa. The static pressure cloud diagram of the impeller fluid domain is shown in Fig. 13c. The maximum negative pressure is 19.8 Pa, near the outlet of the impeller fluid domain; the minimum is − 4183.3 Pa, near the impeller inlet. The dynamic pressure cloud diagram of the fluid domain of the negative pressure cavity is shown in Fig. 13d. The dynamic pressure $p_{dyn}$ is a physical quantity related to the speed, and its specific expression is:

20 $$p_{dyn} = \frac{1}{2}\rho v^{2}$$

From Eq. (20), it can be seen that the dynamic pressure is proportional to the quadratic of the fluid velocity. Because the fluid velocity in the negative pressure cavity is relatively slow, and the overall dynamic pressure is relatively small.

The impeller's dynamic pressure cloud diagram and velocity cloud diagram are shown in Fig. 13e and f, respectively. They are mainly divided into the impeller inlet area, the impeller outlet area, and the transition area between the two. The inlet area is characterized by low dynamic pressure and low flow velocity. In the simulation, the inlet dynamic pressure and velocity are almost 0; the outlet area has high dynamic pressure and high flow velocity. The maximum outlet dynamic pressure is 3380 Pa, and the outlet velocity is 83.1 m/s. As shown in Fig. 13, the transition area of the impeller is between the inlet and the outlet. The dynamic pressure and velocity values in the transition region are not constant. The dynamic pressure value in the transition zone is between the inlet and outlet. The same goes for the velocity values in the transition area.

The total pressure cloud diagram of the fluid area of the negative pressure device is shown in Fig. 13g and h, respectively. The calculation formula of the total pressure $p_{tot}$ in the Fluent is:

21 $$p_{tot} = p_{sta} + p_{dyn}$$

That is, the total pressure is the sum of static pressure and dynamic pressure. It can be seen from Fig. 13g that the total pressure is negative, about 3500 Pa, which is consistent with the static pressure of the negative pressure cavity. It can be seen from Fig. 13h that the impeller negative pressure cavity is divided into three areas: the impeller inlet area with a smaller total pressure, the impeller outlet area with enormous dynamic pressure, and the total pressure are between the transition zone between the inlet and the outlet. It can be seen that for fluids with low flow rates, static pressure plays a significant role in total pressure; for fluids with relatively high flow rates, dynamic pressure plays a significant role in total pressure.

#### Analysis of simulation results of impeller and negative pressure cavity

##### Analysis of simulation results of negative pressure cavity

**Figure 14:**
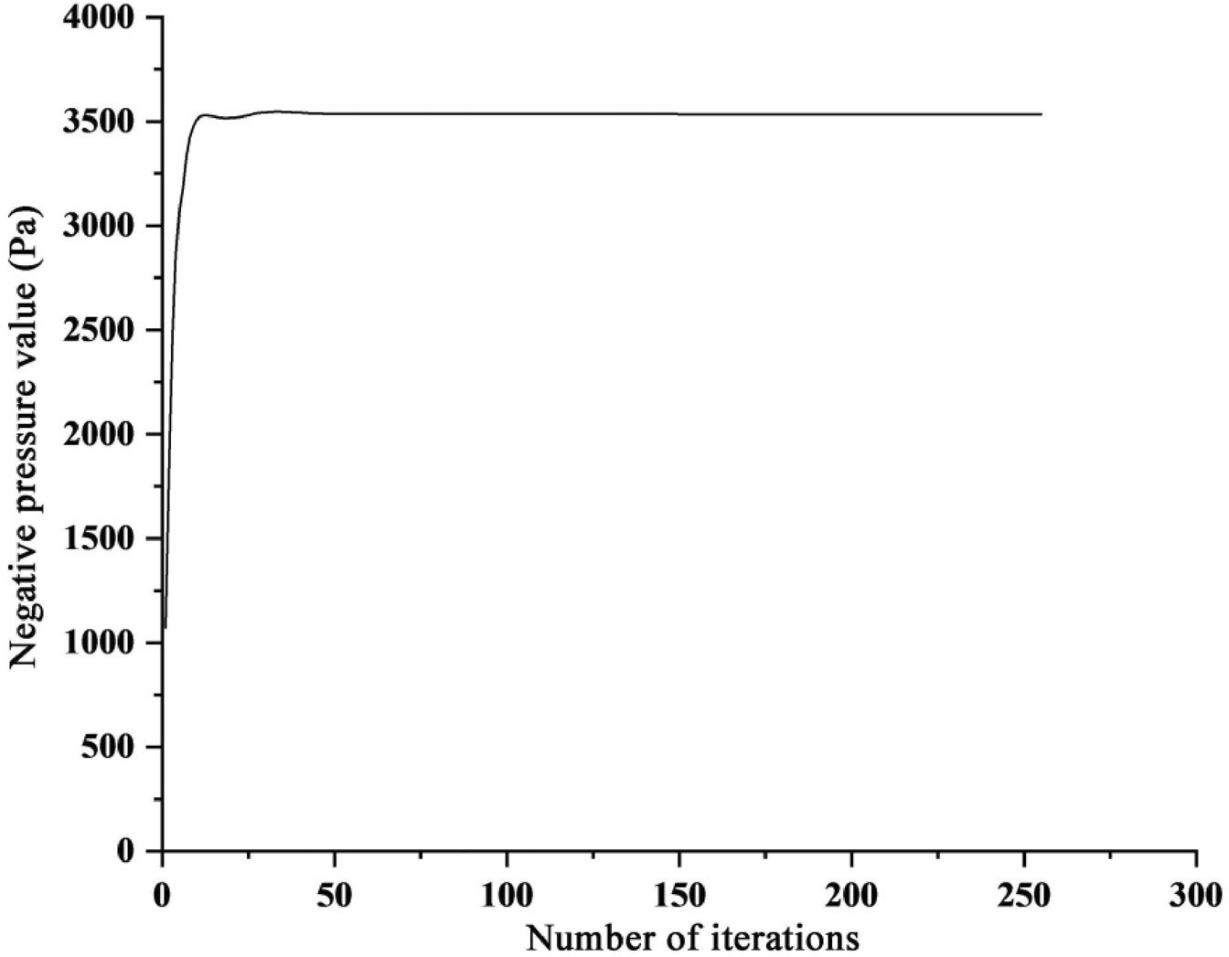
Negative pressure value of negative pressure device.

The change of the negative pressure value at the inlet of the fluid domain of the negative pressure device is shown in Fig. 14. When the number of iteration steps is 1, the calculated inlet negative pressure value is 1072.03 Pa, and then the negative pressure value rises rapidly. When the number of iteration steps is about 50, the negative pressure value reaches a stable state, 3534.75 Pa.

Under the same conditions, multiple simulations were performed on the fluid domain of the negative pressure device, thereby reducing accidental errors caused by the simulation and calculating the negative pressure value of the negative pressure cavity. The simulation results are shown in Table 4:

**Table 4:** Simulation results of negative pressure value.

Simulation times	Negative pressure value (Pa)	Average value (Pa)
1	3534.75	3534.75
2	3534.75	
3	3534.74	

Table 5 lists the negative pressure values of the experimental results and CFD simulation results, and the simulation results are averaged at 3534.75 Pa. For the standard sealing method, the simulation results are consistent with the experimental results, and the error is only 0.23%, which proves the correctness of the simulation calculation results; for the thickened sealing method, the error between the simulation and the experimental results is 5.12%, which is within an acceptable range within. In summary, CFD simulation and actual experiment have a good fit, and CFD simulation can be used instead of experiment.

**Table 5:** Comparison of simulation results and experimental results.

Type	Experimental value (Pa)	Simulation value (Pa)	Error (%)
Sealing method 1	3543.04	3534.75	0.23
Sealing method 2	3727.36		5.12

##### Analysis of the result of impeller simulation total pressure

For an impeller whose outlet is discharged toward the atmosphere, its total pressure equals the total inlet pressure. Table 6 compares the total pressure of the three types of impellers. The first method is to calculate the total pressure difference between the outlet and the inlet of the impeller; the second method is to calculate the total inlet pressure; the third method is to measure it through experiments.

**Table 6:** Comparison of total pressure of impeller.

Method	Total pressure value (Pa)	Design total pressure (Pa)	Error (%)
Method 1	3638.48	5000	27.2
Method 2	3621.93		27.6
Method 3	3604.04		27.9

The comparison shows that the total pressure values obtained by the three methods are not much different, which shows that the CFD simulation results are consistent with the experimental results. However, there is a big gap between the actual total pressure of the impeller and the total design pressure of the impeller. The first is because the surface of the impeller and the blades are relatively rough, and the friction with the air during rapid rotation causes a pressure drop. Second, the design of the impeller is based on an empirical formula. In the process, many parameters are selected based on experience. There is a particular error, which causes a large gap between the total pressure of the impeller and the actual one.

## Optimized design of impeller

### Selection of impeller optimization parameters

The shape of the blade, the size of the inlet of the impeller and the number of blades have an important influence on the performance of the impeller. Therefore, the blade outlet angle, the impeller inlet diameter, and the number of blades are selected as design variables. Table 7 shows the value ranges of the three design variables.

22 $$X = [\beta_{2A} ,D_{0} ,Z].$$

**Table 7:** Design variables and their value ranges.

Design variable	Symbol	Unit	Value range
Blade outlet angle	$\beta_{2A}$	°	[30,70]
Impeller inlet diameter	$D_{0}$	mm	[50,70]
Blade number	$Z$	pcs	[4, 28]

### Experimental design and model selection

Before optimizing the impeller parameters, it is necessary to design them and establish a three-dimensional model for CFD simulation. In order to reduce the number of tests, a specific test method is usually selected for experimental design, which can achieve the expected results in a short time. The BOX design method is a response surface design method proposed by Box and Behnken in 1960 for three or more influencing factors^37^.

**Figure 15:**
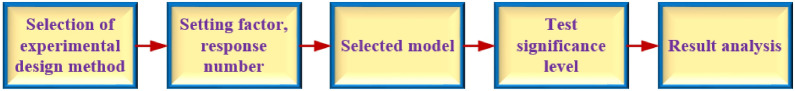
Design-Expert software flow chart.

In order to select appropriate test sample points, the Design-Expert software is used for the relevant design of the BOX method. The Design-Expert software is software specially used for test design and related data analysis. When using it for data analysis, the primary process is as shown in Fig. 15^38–40^. Table 8 shows the sample points generated by the Design-Expert software, with three influencing factors and one dependent variable.

**Table 8:** Selection of sample points and CFD simulation results.

Number	Blade outlet angle (°)	Impeller inlet diameter (mm)	Blade number	Negative pressure value (Pa)
1	30	50	16	2915.483
2	70	50	16	2920.728
3	70	70	16	3652.747
4	30	60	28	2727.532
5	50	60	16	3696.276
6	70	60	4	2929.647
7	50	60	16	3696.276
8	50	60	16	3696.276
9	30	70	16	3542.577
10	30	60	4	2753.91
11	50	60	16	3696.276
12	50	70	4	2762.851
13	50	60	16	3696.276
14	50	70	28	3626.978
15	50	50	28	-462.697
16	50	50	4	2975.21
17	70	60	28	2947.31

### Optimal design of impeller parameters

#### Establish the Kriging model of the impeller

Since there is no apparent functional relationship between the negative pressure value *p* of the impeller evaluation index and the design variables of the impeller, the Kriging model is used to establish the mathematical model of the impeller parameters. The kriging model is a mathematical model with high calculation accuracy, small calculation amount, and short calculation time, which is very suitable for application in optimization. Simpson^41^ and others have proved that the Kriging model has the characteristics of unbiased estimation and nonlinear approximation of sample points. Therefore, the Kriging model is an ideal model for the nonlinear model of the impeller^42^.

The Kriging model is a parameter difference method composed of polynomials and random process parts:

23 $$y\left( {\mathbf{x}} \right) = f\left( {\mathbf{x}} \right)\beta + Z\left( {\mathbf{x}} \right)$$

Among them, $y\left( {\mathbf{x}} \right)$ is an unknown function of interest to the experimenter, $f\left( {\mathbf{x}} \right) = \left[ {f_{1} \left( {\mathbf{x}} \right),f_{2} \left( {\mathbf{x}} \right),...,f_{n} \left( {\mathbf{x}} \right)} \right]^{T}$ is a function of $x$, $\beta = \left[ {\beta_{1} ,\beta_{2} ,...,\beta_{n} } \right]^{T}$ is a correlation coefficient, $Z\left( {\mathbf{x}} \right)$ is a random error function with a mean value of 0, and a variance of not 0, that is:

24 $$\left\{ \begin{gathered} E\left[ {Z\left( {\mathbf{x}} \right)} \right] = 0 \hfill \\ Var\left[ {Z\left( {\mathbf{x}} \right)} \right] = \sigma^{2} \hfill \\ \end{gathered} \right.$$

And its covariance is:

25 $$Cov\left[ {Z\left( {{\mathbf{x}}^{i} } \right),Z\left( {{\mathbf{x}}^{j} } \right)} \right] = \sigma^{2} {\mathbf{R}}\left( {{\mathbf{x}}^{i} ,{\mathbf{x}}^{j} } \right)$$

Among them, **R** $\left( {{\mathbf{x}}^{i} ,{\mathbf{x}}^{j} } \right)$ is a $n \times n$ correlation matrix that is symmetrical along the diagonal, and ${\mathbf{R}}\left( {{\mathbf{x}}^{i} ,{\mathbf{x}}^{j} } \right)$ is the correlation function between any two observation points $x^{i}$ and $x^{j}$ on the diagonal elements. The related function ${\mathbf{R}}\left( {{\mathbf{x}}^{i} ,{\mathbf{x}}^{j} } \right)$ is:

26 $$R({\mathbf{x}}^{i} ,{\mathbf{x}}^{j} ) = exp\left[ { - \sum\limits_{k = 1}^{N} {\theta_{k} \left| {x_{k}^{i} - x_{k}^{j} } \right|^{2} } } \right]$$

Among them, *N* is the number of design variables, $\theta_{k}$ is an unknown related parameter, $x_{k}^{i}$ and $x_{k}^{j}$ are the *k*-th component of the observation points $x^{i}$ and $x^{j}$ respectively. The estimated value $\hat{y}(x^{*} )$ at the unknown point $x^{*}$ is:

27 $$\hat{y} = \hat{\beta } + {\mathbf{r}}^{T} (x^{*} ){\mathbf{R}}^{ - 1} ({\mathbf{y}} - p\hat{\beta })$$

where $y = \left[ {y_{1} ,y_{2} ,...,y_{n} } \right]^{T}$ is the response value of $f\left( x \right)$. When $f\left( x \right)$ is a constant, **p** is a column vector containing components $n_{0}$, and:

28 $$\left\{ \begin{gathered} \hat{\beta } = ({\mathbf{p}}^{T} {\mathbf{R}}^{ - 1} {\mathbf{P}}){\mathbf{p}}^{T} {\mathbf{R}}^{ - 1} {\mathbf{y}} \hfill \\ {\mathbf{r}}^{T} (x^{*} ) = [{\mathbf{R}}(x^{*} ,x^{1} ),{\mathbf{R}}(x^{*} ,x^{2} ),...,{\mathbf{R}}(x^{*} ,x^{{n_{o} }} )] \hfill \\ \end{gathered} \right.$$

By maximizing the following expression, the maximum likelihood estimate $\theta_{k}$ in the equation can be obtained:

29 $$\mathop {\max }\limits_{\theta > 0} \frac{{ - [n_{0} \ln (\hat{\sigma }^{2} ) + \ln ({\mathbf{R}})]}}{2}$$

Use the sample data in Table 8 to establish a kriging surrogate model between impeller design parameters and negative pressure values. The negative pressure value corresponding to the original design parameters is predicted using the established kriging model and compared with the original data. The comparison results are shown in Table 9. The predicted value of negative pressure value of the kriging model is 3498.55 Pa, and the error with the simulation result is only 1.02%, which proves the correctness of the impeller kriging model.

**Table 9:** Kriging model prediction value and original design verification.

Type	$\beta_{2A}$(°)	$D_{0}$(mm)	Z	Negative pressure value (Pa)
Original design	50	62	10	3534.75
Predicted value				3498.55
Error	–	–	–	1.02%

#### Genetic algorithm optimization

Genetic algorithm (GA) is an optimization algorithm based on the evolutionary theory of "natural selection by nature, survival of the fittest." It mainly involves operations such as selection, crossover, and mutation. The higher the fitness of the individual, the greater the probability of reproducing offspring. On the contrary, if the individual's fitness is low, the probability of reproducing offspring is low, or even weeding out offspring without reproducing. Therefore, the optimal generation can be obtained through such continuous genetic optimization^43^.

According to the CFD simulation results, the negative pressure value *p* of the negative pressure cavity is selected as the evaluation index of the impeller parameters. The larger the negative pressure value, the better, so the objective function of impeller optimization can be expressed as:

30 $$f(X) = - p$$

**Figure 16:**
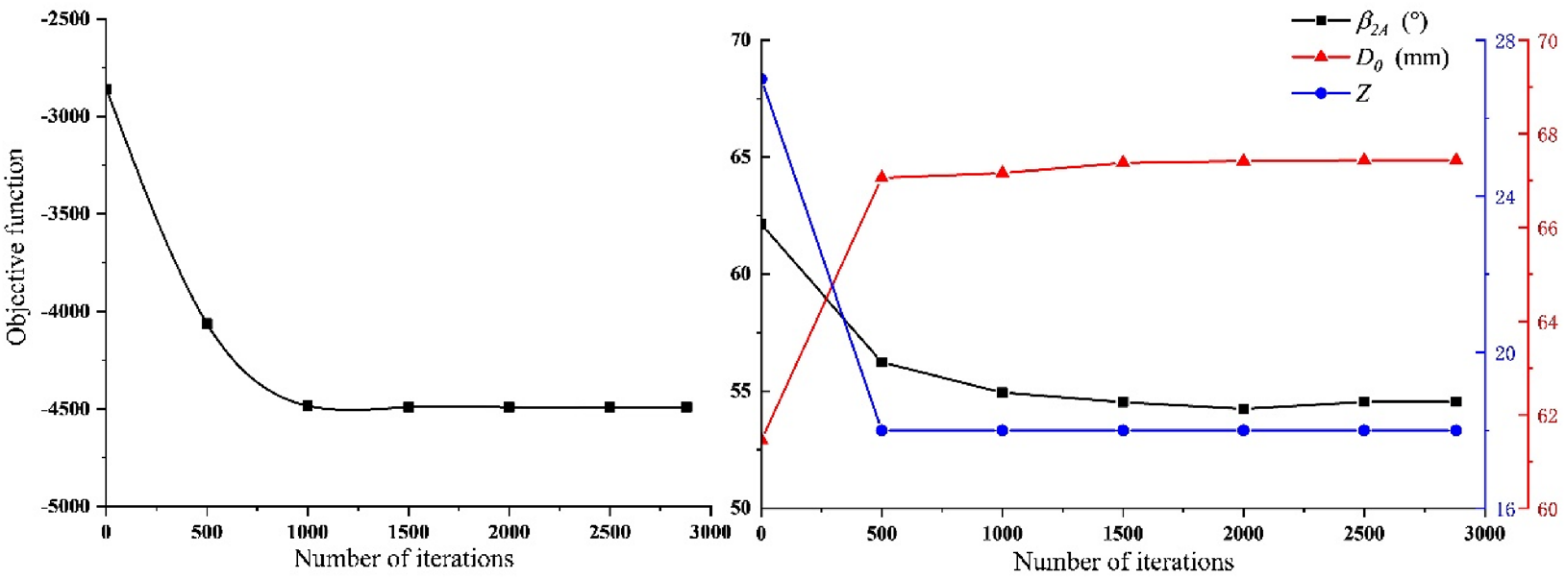
Optimization of objective function and design variables.

Using GA to optimize the design of the established optimization model, the value of the objective function and the optimization process of the three design variables are shown in Fig. 16.

It can be seen from Fig. 16 that the convergence value of the objective function is − 4491.19, the convergence value of the blade outlet angle is 54.55°, the convergence value of the impeller inlet diameter is 67.43 mm, and the convergence value of the number of blades Z is 18. The optimized result is the same as the original design. The comparison is shown in the Table 10. Compared with the original design, the negative pressure value has been increased from 3534.75 Pa to 4491.19 Pa, increasing 27.06%. Therefore, the optimized design results of the impeller parameters are: the blade outlet angle is 54.55°, the impeller inlet diameter is 67.43 mm, and the number of blades Z is 18. Under this set of parameters, the corresponding negative pressure value is 4491.19 Pa.

**Table 10:** Comparison of optimization results.

Type	$\beta_{2A}$(°)	$D_{0}$(mm)	Z	Negative pressure value (Pa)
Original design	50	62	10	3534.75
Predicted value	54.55	67.43	18	4491.19

## Conclusion

This paper takes the negative pressure adsorption wall-climbing robot as the research object. Through CFD simulation and prototype experiment, the influence of wall material, adsorption position, shape of the negative pressure cavity and sealing method on the adsorption performance of the wall-climbing robot is studied, and it is based on Kriging. The model and genetic algorithm optimize the parameters of the impeller in the adsorption device, and the following conclusions are obtained:

1. Without considering the deformation of the wall surface, the wall material has little effect on the adsorption performance.
2. By comparing the negative pressure value when the robot is adsorbed in three different poses, it can be considered that the posture of the wall-climbing robot does not affect the negative pressure value.
3. The shape and sealing method of the negative pressure cavity have an essential impact on the adsorption performance of the wall-climbing robot. By changing the structure of the sealing edge and the structure of the negative pressure cavity, the adsorption capacity can be improved.
4. The experiment verifies the correctness of the CFD simulation model. The method of obtaining sample points based on CFD model simulation is more stable and saves time. Compared with the original design, the impeller parameters optimized based on the kriging method and GA have increased the negative pressure value from 3534.75 to 4491.19 Pa, an increase of 27.06%.

## Supplementary Information

Supplementary Information.

## Data Availability

All data are available in the main text or the supplementary materials.
