# Supplementary material for: Design and optimization of wall-climbing robot impeller by genetic algorithm based on computational fluid dynamics and kriging model
Source: Sci Rep. 2022 Jun 10;12:9571. doi: 10.1038/s41598-022-13784-z (PMC9187699; doi:10.1038/s41598-022-13784-z)
Supplement: Supplementary file 1 — Supplementary Information. [file 41598_2022_13784_MOESM1_ESM.docx]

**Appendix 1: Impeller parameter design method**

Incorporating the parameters of Table 1 into equation (19), the adsorption condition of the wall-climbing robot is: $F_{p}>223\text{ N}$, then the minimum adsorption pressure required by the wall-climbing robot is:

$p_{\min}=\frac{F_{p}}{l_{1}l_{2}}=\frac{223}{0.25\times0.2}\text{ Pa=4460 Pa}$ （31）

Under the premise of ignoring the pressure loss of the negative pressure along the height direction of the negative pressure chamber, the minimum adsorption pressure required by the negative pressure chamber is the total pressure of the impeller. According to the calculated minimum adsorption pressure, the working pressure of the impeller is selected as 5000 Pa, and the initial design parameters of the impeller are shown in Table 10.

**Table 10 Initial design parameters of impeller**

| **Parameter** | **Symbol** | **Value** | **Unit** |
| --- | --- | --- | --- |
| Full pressure | *p* | 5000 | Pa |
| Flow rate | *q* | 0.03 | m^3^/s |
| Rotate [speed](file:///C:\Users\MrBigDong\AppData\Local\youdao\dict\Application\7.5.0.0\resultui\dict\?keyword=speed) | *n* | 8000 | r/min |

According to the initial design parameters of the impeller, other design parameters can be calculated. The specific speed of the impeller $n_{s}$ is:

$n_{s}=5.54n\frac{q^{\frac{1}{2}}}{p^{\frac{3}{4}}}=5.54\times8000\times\frac{0.03^{\frac{1}{2}}}{5000^{\frac{3}{4}}}=12.91$ （32）

Because the specific speed is relatively small, the backward curved arc blade is selected. The exit angle of the primarily selected blade is $\beta_{2A}\text{=}50^{\circ}$. The estimated total pressure coefficient $\psi_{t}$ is:

$\begin{matrix} \psi_{t}=\left(0.2835+2.7966\times10^{-3}\beta_{2A}-1.439\times10^{-5}n_{s}^{2}\right)\times2 \\ =\left(0.2835+2.7966\times10^{-3}\times50-1.439\times10^{-5}\times12.91^{2}\right)\times2=1.04 \end{matrix}$ （33）

The outer peripheral speed of the impeller $u_{2}$ is:

$u_{2}=\sqrt{\frac{2p}{\rho\psi_{t}}}=\sqrt{\frac{2\times5000}{1.20\times1.04}}\text{ m/s}=89.51\text{ m/s}$ （34）

The outer diameter of the impeller $D_{2}$ is:

$D_{2}=\frac{60u_{2}}{\pi n}=\frac{60\times89.51}{3.14\times8000}\text{ m}=0.213\text{ m}$ （35）

Take $D_{2}=0.200\text{ m}$, then $u_{2}=83.73\text{ m/s}$. The corrected total pressure coefficient $\psi_{t}$ is:

$\psi_{t}=\frac{2p}{\rho u_{2}^{2}}=\frac{2\times5000}{1.20\times83.73^{2}}=1.19$ （36）

The flow coefficient is:

（37）

The specific diameter of the impeller $D_{s}$ is:

（38）

Taking the impeller inlet speed $c_{0}=10\text{ m/s}$, the impeller inlet diameter $D_{0}$ and the blade inlet diameter $D_{1}$ are:

$D_{0}=\sqrt{\frac{4q}{\pi c_{0}}}=\sqrt{\frac{4\times0.03}{3.14\times10}}\text{ m=0.062 m}$ （39）

$D_{1}=1.1D_{0}=1.1\times0.062\text{ m}=0.068\text{ m}$ （40）

Then $D_{1}/D_{2}=0.341$, take the air intake speed before the blade entrance$c'_{1}=1.1c_{0}=1.1\times10\text{ m/s}=11\text{ m/s}$. Then the airflow angle before the blade entrance $\beta'_{1}$ is:

$\tan\beta'_{1}=\frac{c_{1}}{u_{1}}=\frac{11\times60}{3.14\times0.0682\times8000}=0.3852$ （41）

Therefore $\beta'_{1}\text{=}21^{\circ}7'$, taking the angle of attack $i'=8^{\circ}53'$, the geometric angle of the blade entrance $\beta_{1A}$ is:

$\beta_{1A}=21^{\circ}7'+8^{\circ}53'=30^{\circ}$ （42）

The blade entrance width $b_{1}$ is:

$b_{1}=\frac{q}{\pi D_{1}c'_{1}}=\frac{0.03}{3.14\times0.0682\times11}\text{ m=0.0127 m}$ （43）

Take $b_{1}=0.013\text{ m}$, the number of leaves $Z$ is:

$Z=8.5\frac{\sin\beta_{2A}}{1-\frac{D_{1}}{D_{2}}}=8.5\frac{\sin50^{\circ}}{1-0.341}=9.88$ （44）

Take $Z=10$, the width of the blade outlet $b_{2}$ is:

$b_{2}=\frac{b_{1}D_{1}}{D_{2}}=0.0127\times0.341\text{ m}=0.0043\text{ m}$ （45）

Pick $b_{2}=0.004\text{ m}$.
